# Prospective, randomized, double-blinded, placebo-controlled study on safety and tolerability of the krill powder product in overweight subjects with moderately elevated blood pressure

**DOI:** 10.1186/s12944-018-0935-x

**Published:** 2018-12-20

**Authors:** Essi S. Sarkkinen, Markku J. Savolainen, Jyrki Taurio, Tuuli Marvola, Inge Bruheim

**Affiliations:** 1 Food and Nutrition, Oy Medfiles Ltd (CRO), P.O.Box 1450, 70701 Kuopio, Finland; 2 Research Unit of Internal Medicine, Medical Research Center Oulu, Oulu University Hospital and University of Oulu, P.O.Box 5000, 90014 Oulu, Finland; 3 Department of Internal Medicine, University of Tampere and FinnMedi Oy, 33014 Tampere, Finland; 4 Oy Medfiles Ltd (CRO), P.O.Box 1450, 70701 Kuopio, Finland; 5 Rimfrost AS, Skansekaia 3 C, Ålesund, 6002 Norway

**Keywords:** Krill powder, Krill oil, Safety, Tolerability, Adverse event, Omega-3 fatty acids, Eicosapentaenoic acid, Docosahexaenoic acid

## Abstract

**Background:**

Krill powder is rich in bioactive ingredients such as eicosapentaenoic acid (EPA), docosahexaenoic acid (DHA), phospholipids, protein and astaxanthin. Containing dominantly EPA, it is considered to be effective in lowering lipids, foremost serum triglycerides and LDL cholesterol. Krill-derived protein hydrolysates/peptides may have positive effect on blood pressure and astaxanthin has anti-oxidative and anti-inflammatory properties. Thus, krill powder has a lot of potential in improving lipid and metabolic profile and reinforcing the activity of the antioxidant system. However, randomized clinical trials on krill powder are scarce and systematic data of krill meal on human safety is limited. Some of the earlier studies have reported several, non-serious adverse events, mostly related to gastrointestinal tract, but systematic sufficiently powered study on safety is lacking. The aim of this study was to collect data on safety and tolerability of krill powder in humans and simultaneously gain efficacy data by measuring the risk factors for cardiovascular disease.

**Methods:**

The study was a randomised, double-blinded, placebo-controlled intervention study with 35 overweight subjects with mildly or moderately elevated blood pressure, who took 4 g krill oil powder or 4 g of placebo during an 8-week follow-up period. The study consisted of a pre-screening, screening, day 0 baseline (randomization visit) and three follow-up visits on days 14, 28 and 56. The reported adverse events in the groups were compared as primary endpoint and haematological safety parameters and changes in systolic and diastolic pressure and blood total and lipoprotein lipids were measured as secondary end points.

**Results:**

There were in total 80 reported adverse events during the follow-up; 50 in placebo and 30 in krill powder group. Gastrointestinal symptoms (flatulence, heartburn and diarrhea) were the most commonly reported among those probably related to the test products. No serious adverse events were reported. The mean value of all measured hematology variables remained within the reference values in all study subject and no significant changes were observed in blood pressure or lipid values.

**Conclusions:**

The results seem to indicate that using krill powder as a source for EPA and DHA is safe in therapeutic dose and the risk of adverse events, let alone serious ones, is low.

**Trial registration:**

ClinicalTrials.gov, NCT03112083, retrospectively registered.

## Background

Krill powder is reddish powder produced from antarctic krill (*Euphasia superba*). It is rich in multiple bioactive ingredients such as EPA and DHA, phospholipids, protein and astaxanthin. Krill oil has been studied repeatedly but the studies on krill meal and its safe use in humans are few.

Krill oil contains dominantly EPA compared to fish oils containing higher proportion of DHA [1]. The effect of long-chain n-3 fatty acid on serum LDL cholesterol is slightly controversial, since the effect has been found to be neutral or even adverse with some formulations or high doses and in some studies. In general, treatment with EPA + DHA appears to lower patient triglycerides effectively, but in patients with very high triglyceride levels, use of EPA + DHA may raise low-density lipoprotein cholesterol levels, whereas EPA alone may not do that. It is well established that triglyceride concentrations are linked to other markers of metabolic syndrome such as insulin [1–3]. The effect of krill powder on non-lipid metabolic markers such as blood glucose is much more controversial and human studies have been mostly made with krill oil, only one with krill meal so far [4–8].

EPA and DHA are known to increase hemostasis via their inhibitory effects on platelet function, without increasing bleeding as such [1, 9] . EPA and DHA also suppress the production of arachidonic-acid-derived eicosanoids and EPA is a substrate for the synthesis of an alternative family of eicosanoids. Thus, dietary fats which are rich in n-3 PUFAs have the potential to alter cytokine production and modulate inflammation [5, 6]. In addition, astaxanthin is a carotenoid that contributes to krill’s anti-oxidative and anti-inflammatory properties [7]. However so far, the in vivo data in humans with krill meal derived astaxanthin are also scarce [10, 11].

Recently more and more interest has also been paid to the cardio-protective activity of proteins or peptides derived from marine sources [12]. Marine proteins and peptides could have a lowering effect on blood pressure [11, 13, 14]. Krill powder is relative high in protein (55%) and therefore it is interesting to evaluate its effect on blood pressure and other vascular elasticity markers, especially since current human data on antihypertensive activity of krill-derived protein hydrolysates/peptides is lacking also in safety terms [14–16].

In summary, krill powder with its various bioactive components has a lot of potential in improving lipid profile, suppressing lipid peroxidation, and reinforcing the activity of the antioxidant system and thus it reduces vascular wall inflammation, stabilizes membrane properties or increases vascular elasticity. These all together could prevent progression of atherosclerosis. However, as randomized clinical trials on krill powder are still scarce [7, 17], systematic data on human safety is limited sofar. Some of the earlier studies have reported several, non-serious adverse events, mostly related to gastrointestinal tract [7, 17], but no systematic study focusing on safety has been done yet. Consequently, there was a clear need to systematically collect data on its safety and tolerability in humans. Simultaneously, we aimed to gain efficacy data on krill powder by measuring various risk factors for cardiovascular disease.

## Study objectives

### Primary objective

The primary objective of the study was to compare the amount and the type of adverse events during 8-week follow-up after ingestion of krill powder preparation in comparison to ingestion of respective amount of placebo in overweight study subjects with mildly or moderately elevated blood pressure.

### Secondary objectives

In addition to adverse effect, the aim was also to compare safety parameters, i.e. blood count, creatinine, gamma glutamyl transferase, aspartate aminotransferase (AST) and (alanine aminotransferase) ALT and to measure changes in selective efficacy parameters such as systolic and diastolic blood pressure as well as blood total and lipoprotein lipids during the whole 8-week follow-up period of the study.

### Subjects and methods

#### Study subjects

Study subjects were recruited through advertisements in local newspapers, public notice boards and research sites from Tampere and Oulu area (in Central and Northern Finland). Inclusion criteria were: 1) age 18–65 years, 2) overweight female or male (BMI between 25 and 30 kg/ m^2^), 3) mildly or moderately elevated blood pressure (systolic 140–159/ diastolic 90–99 mmHg) and 4) signed written informed consent. The exclusion criteria were: 1) medication potential to affect serum lipids (lipid-lowering drugs), 2) familial hypercholesterolemia, marked combined hyperlipidemia, condition that would impair fat absorption (e.g. chronic pancreatitis, pancreatic lipase deficiency syndrome), 3) any untreated medical condition affecting absorption of fat, 4) type 1 and 2 diabetes, 5) cancer or other malignant disease within the past 5 years, 6) periodical hormone replacement therapy, 7) high intake of oily fish (> 2 times per week as a principal meal) (i.e. salmon, herring, sardines, mackerel, vendace), 8) smoking, 9) alcohol consumption > 15 doses per week, 10) pregnant, lactating or wish to become pregnant, 11) hypersensitivity to fish or any of the components of the test products, 12) regular use (> 3 times per week) of n-3 or other fatty acid supplements, plant sterols or fiber supplements 4 weeks before randomization, 13) lack of suitability for participation in the trial, for any medical reason, as judged by the PI.

Subjects would have been withdrawn from the study if pregnancy or any other diagnosed medical condition or treatment stated above as the exclusion criteria had taken place during the study. All study subjects had a right to discontinue the study at any point, if they wanted to stop taking part in the study.

#### Study design and procedures

The study was a randomised, double-blinded, placebo-controlled intervention study with overweight subjects with mildly or moderately elevated blood pressure. Two-arm parallel design was followed in the study. Study was conducted at two study sites in Central (Tampere) and Northern Finland (Oulu). In total 35 subjects were randomised according to randomisation list to two groups (krill powder or placebo) in a balanced manner (1:1), separately for both gender and site. Concealed allocation was used to keep both subjects and staff blinded. The study consisted of a pre-screening, a screening visit 7 to 14 days before randomization, Day 0 baseline (Randomization visit) and 8-week intervention with safety and tolerance follow-up period with three visits on Day 14, Day 28 and Day 56.

A total of 6 study visits were included. At pre-screening visit the study subjects were requested to sign informed consent form. A structured interview on demographics (age, sex, ethnicity), previous and current diseases, current medication, alcohol and tobacco consumption and use of dietary supplements (especially fish oil and other n-3 FA supplements, plant sterols and cholesterol lowering fibre supplements (guar gum, glucomannan, oat fibre etc.) and use of fish foods was carried out at the screening visit and replicated at the day 56 visit.

At baseline visit primary (number of adverse events) and all secondary and optional endpoint measurements were taken. At day 14, 28 and 56, the study diaries were checked and blood pressure was measured. During day 28 and 56 visits safety laboratory measurements and blood lipoprotein measurements were also taken. At day 56 the test product accountability was performed to check the compliance during the follow-up.

#### Test products and background diet

Study included one test product: krill powder derived from antarctic krill (*Euphausia superba)* (Rimfrost Pristine®, Rimfrost AS, PO box 234, 6099 Fosnavåg, Norway) and placebo product and both were given in capsule form, 4 capsules in the morning and 4 in the evening providing 4 g dose daily. Maize starch (Pharmatech AS, Rolvsøy, Norway) acted as an inert placebo and was filled in the parallel capsules as the krill powder. Same excipients and masking flavour were used in krill and placebo capsules. Composition for active product is given in the Table 1. Study products were delivered to study subjects at Day 0, Day 14 and Day 28 visits equaling the amounts needed for each follow-up period with extra capsules for 1 week. Study personnel recorded the amounts of capsules given for each study subject and the amount returned (also empty blisters) at each visit.

**Table 1:** Composition of krill powder Rimfrost Pristine®

Nutrient	Amount	Unit
Moisture	max 6	%
Fat	max 25	%
Protein (N*6.25)	min 55	%
Ash	max 10	%
Phopsholipids	min 10	%
EPA (20:5 n-3) as FFA	min 2.5	g/100 g
DHA C22:6 n-3 as FFA	min 1.5	g/100 g
Total n-3 as FFA	min 5.0	g/100 g
Esterified Astaxanthin	min 40	mg/kg

Nutritional counselling regarding the consumption of fish, omega-3 and -6 fatty acids, food supplements and investigational product for the duration of the study were given for the study subjects at the screening visit by a study nurse or registered dietitian and compliance was followed throughout the study. The subjects were advised to keep their medication, lifestyle, background diet and body weight constant during the study and deviation were recorded into the diary.

#### Primary endpoint: Adverse event (AE) follow-up

As a primary endpoint of the study, the total number of reported adverse events were compared in the study subject groups taking 8 capsules (4 g) krill oil powder or 8 capsules (4 g) of placebo for the 8-week follow-up period. Any unfavourable and unintended sign, symptom or medical complaint and worsening of a pre-existing condition was regarded as AE. Study subjects kept diary for the whole duration of the study and were requested to write down all unfavourable symptoms and medical complaints not existing at baseline or significantly worsened from baseline situation. Completeness of diaries was checked at each study visit. All reported adverse events were recorded, coded and analysed carefully to determine severity, possible relation to study products, onset and outcome of the event.

#### Anthropometric and blood pressure measurements

Body weight was measured with the same calibrated, digital scale at screening visit and at Day 56 follow-up visit while the subjects were wearing light indoor clothing and no shoes and recorded with 0.1 kg precision. The weight measurement was replicated once and the mean of two measurements was used in the statistical analysis. Body mass index was calculated with the following formula: body mass (kg) / (height (m))^2^. Height was measured at screening visit with the subject standing straight, hands on the side, shoulders relaxed and heels together in so called Frankfurt position and result was recorded to the nearest 0.5 cm.

Blood pressure (BP) was measured at all visits using automatic blood pressure measuring device that was validated according to standardized protocols and calibrated periodically. Before BP measurements, study subjects were advised to avoid heavy physical exercise, smoking and consumption of caffeine rich drinks. BP measurements were conducted in a quiet room and subjects were seated comfortably, with back supported, legs un-crossed, and subjects were allowed to rest 3–5 min, cuff assembled, before starting the BP measurement. Blood pressure was measured from the upper arm, supported at the hearth level. Proper size cuff was placed to the upper arm so that middle part of the bladder was on the brachial vein. Three BP measurements spaced 1–2 min apart were taken and recorded with 1 mmHg accuracy. In case first two measures differed significantly (over 10 mmHg), additional measurements were taken. Average of last two measurements were used in the analysis. At the first visit measurements were taken repeatedly from both arms and if the measures differed significantly, in the follow-up measurements the arm giving higher values was used.

#### Laboratory measurements

Study subjects were instructed to follow normal fasting procedures before the study visits if blood samples were taken, i.e. to be without food and drink (except small amount of water) 10–12 h before sampling and to avoid alcohol consumption also the day before laboratory tests. Routine clinical chemistry and hematology (blood count, serum thyrotropin, serum creatinine, plasma gamma-glutamyl transferase, blood glucose, AST, ALT) were analyzed at Day 0 and 56 with standardized clinical chemistry and hematology methods at Fimlab or Medix Laboratories. Plasma total and lipoprotein lipids were analysed using commercial methods and reagents; total triglycerides and total cholesterol with enzymatic, colorimetric test and LDL- and HDL-cholesterol concentrations with homogenous enzymatic colorimetric method at Fimlab or Medix Laboratories (Cobas 6000 analyzer (c501 module), Roche Diagnostics, Germany or ADVIA 1800 System, Siemens Healthcare Inc., NY USA).

#### Statistical method

The primary variable, number of reported adverse events in the study groups was compared descriptively and using a Poisson regression model suitable for analysing count data. Safety laboratory and metabolic variables between the study groups were compared using an analysis of covariance models (ANCOVA) adjusting for baseline values. Significance level was set to *p* < 0.05.

## Results

A total of 35 subjects signed informed consent and were randomized according to the randomization list to two groups (krill powder or placebo). Out of the study subjects 18 were male and 17 female and the mean age was 55.4 ± 8.6 years*.* All participants were Caucasian and their mean blood pressure at the screening was 140.0 ± 8.9 /87.6 ± 7.6 mmHg. The mean BMI at the screening was 28.7 ± 1.3 for the krill powder group and 27.3 ± 1.3 for the placebo group, and 28.9 ± 1.4 and 27.7 ± 1.4 at day 56 subsequently. Respective values at baseline are provided in the Table 2.

**Table 2:** Baseline characteristics of the study subjects

		ALL *n* = 35	Krill Powder Value *n* = 18	Placebo Value *n* = 17
Gender (n)	Female	17	8	9
	Male	18	10	8
Race (n)	Caucasian	35	18	17
Age (yrs)	N	35	18	17
	Mean	55.4	55.3	55.5
	SD	8.6	8.4	9.0
	Min	33	38	33
	Median	57.0	57.5	57.0
	Max	65	65	65
BMI (kg/m^2^)	Mean	28.0	28.7	27.3
	SD	1.3	1.3	1.3
Systolic blood pressure (mmHg)	Mean	138.7	141.8	135.4
	SD	8.5	8.7	7.2
Diastolic blood pressure (mmHg)	Mean	87.2	88.6	85.6
	SD	6.9	7.7	5.8
Total cholesterol (mmol/l)	Mean	5.79	5.78	5.79
	SD	0.89	0.80	1.00
LDL cholesterol (mmol/l)	Mean	3.92	3.98	3.86
	SD	0.92	0.94	0.92
HDL cholesterol (mmol/l)	Mean	1.56	1.49	1.63
	SD	0.48	0.58	0.34
Triglycerides (mmol/l)	Mean	1.30	1.31	1.29
	SD	0.54	0.49	0.61

*BMI* body mass index, *HDL* high density lipoprotein, *LDL* low density lipoprotein

### Safety

Reported adverse events were coded according system organ class following adapted ICD-10 main classes. All safety parameters were measured at screening, base line, day 28 and day 56 and all adverse events reported by the subjects in their study diaries since the last visit were collected at day 14, 28 and 56.

### Adverse events in main organ classes

There were in total 80 reported AEs during the 56-day follow-up in 35 subjects (Table 3). Out of those reported 50 were in placebo group and 30 in krill powder group respectively. The most common AEs reported were gastrointestinal, respiratory (including common cold), musculoskeletal (including injuries) and nervous system (including headache). Out of the 80 reported 12 were classified to be possibly related to the study products and only one probably (heartburn in the krill powder group) and none of the AEs were classified to be definitely related to the study products*.* Gastrointestinal symptoms such as flatulence, heartburn and diarrhea, were the most commonly reported among those possibly related to the test products. No serious adverse events were reported during the study.

**Table 3:** Adverse events by adapted ICD10 main class, number of subjects (N) and event count (F) in all study subjects during the whole intervention period

Classified Event	All			Krill Powder			Placebo		
	F	N	%	F	N	%	F	N	%
Total^a^	80	26	74.3	30	10	55.6	50	16	94.1
Gastrointestinal	23	13	37.1	11	5	27.8	12	8	47.1
Mental, behavioral	2	2	5.7	–	–	–	2	2	11.8
Musculosceletal (inc. injuries)	15	9	25.7	4	3	16.7	11	6	35.3
Nervous system (inc. headache)	14	7	20.0	8	3	16.7	6	4	23.5
Other (inc. unclassified abnormal lab values)	6	5	14.3	–	–	–	6	5	29.4
Respiratory (inc. common cold)	20	15	42.9	7	5	27.8	13	10	58.8

^a^Rate ratio (active/placebo) of adverse event counts (poisson regression model), contrast estimate results: mean (0.5667; Confidence limits 0.3604; 0.8911)

### Hematology and clinical chemistry variables

The mean value of all measured hematology variables remained within the reference values in all study subject both in placebo and treatment group and there were no marked differences between the groups in hematology variables during the intervention. There were no major differences between the groups or in comparison to the baseline readings in the values describing liver and kidney function (Table 4). Serum creatinine (S-Krea) and serum enzyme glutamyl transferase (S-GT) values remained well within the reference values throughout the study. AST remained unchanged at group level, but there was a clear baseline difference (attributed mainly to one outlier) between the groups which slightly diminished during the intervention. ALT value increased markedly in placebo group and this change was attributed mainly to one subject having markedly higher ALT and AST values at day 56 visit.

**Table 4:** Liver and kidney function values at screening, baseline, day 28 and day 56

Parameter	Krill powder, mean value (*n* = 18)	Krill powder, SD	Placebo, mean value (*n* = 17)	Placebo, SD	*P*-value (Krill powder vs. Placebo)
S-ALT (U/I)					
SCR	25.4	12.0	30.5	14.1	
BAS	25.8	12.0	32.6	20.9	
D28	27.5	14.9	30.0	18.3	0.1231
D56	27.1	9.0	41.0	33.5	0.2297
S-AST (U/I)					
SCR	23.6	4.5	26.4	5.5	
BAS	24.9	5.8	36.1	38.3	
D28	27.4	12.5	34.3	37.8	0.2356
D56	24.3	4.8	34.9	23.9	0.1521
S-GT (U/I)					
SCR	26.0	11.1	29.1	13.7	
BAS	27.0	14.3	28.5	13.7	
D28	26.2	10.8	28.1	12.5	0.4191
D56	27.7	11.3	30.1	14.2	0.4160
S-Krea (μmol/L)					
SCR	73.9	10.6	75.8	12.4	
BAS	73.5	9.5	75.5	13.6	
D28	72.2	12.0	74.6	12.3	0.7920
D56	72.5	10.9	74.7	14.9	0.9478

*SCR* screening, *BAS* baseline, *S- ALT* serum alanine aminotransferase, *S-AST* serum aspartate transaminase, *S-GT* serum gamma glutamyl transferase, *S-Krea* serum creatinine

No significant changes were observed in any of the other measured hematology and clinical chemistry values during the whole intervention in either of the groups (Table 5).

**Table 5:** Hematological values at screening, baseline, day 28 and day 56

Parameter (unit)	Krill powder, mean value (*n* = 18)	Krill powder, SD	Placebo, mean value (*n* = 17)	Placebo, SD	*P*-value (Krill powder vs. Placebo)
B-Hb (g/L)					
SCR	147.8	11.2	146.1	8.4	
BAS	146.2	10.2	144.2	8.1	
D28	147.4	11.2	145.1	8.7	0.8074
D56	145.6	11.6	143.8	7.3	0.9517
B-HCT (%)					
SCR	44.3	2.9	43.9	2.1	
BAS	43.4	2.8	42.8	2.5	
D28	44.5	2.4	44.3	2.2	0.7642
D56	44.1	3.2	43.1	2.3	0.3581
E-MCV (fL)					
SCR	90.1	4.7	88.5	4.6	
BAS	89.5	4.7	87.2	4.2	
D28	90.8	4.7	89.6	5.4	0.2584
D56	90.8	4.9	88.7	5.2	0.9008
E-MCH (pg)					
SCR	30.1	2.3	29.5	1.8	
BAS	30.2	2.1	29.5	1.9	
D28	30.1	2.4	29.5	2.0	0.6181
D56	30.1	2.2	29.7	2.0	0.1709

*SCR* screening, *BAS* baseline, *B-Hb* bood hemoglobin, *B-HCT* blood hematocrit, *E-MCV* erythrocyte mean corpuscular volume, *E-MCH* mean corpuscular hemoglobin

### Other results

In addition to safety parameters, LDL, HDL and total cholesterol as well as triglyceride values were also measured in all study subjects at baseline, 28 and 56 days and blood pressure at baseline, 14, 28 and 56 days, as these were considered interesting from the efficacy point of view.

### LDL, HDL, total cholesterol and triglycerides

No significant change was observed in LDL, total cholesterol or triglyceride values in either of the groups during the intervention, *p*-values for within change were 0.9114, 0.7129 and 0.6410 in the placebo group and 0.8190, 0.8221 and 0.1818 in the krill powder group respectively. However, a significant increase was found in the HDL values in the placebo group at the end of the intervention (*p* = 0.0231) but not in the krill powder group (*p* = 0.7053). No significant difference was observed in any of the values at any of the timepoints when comparing the two groups to each other. Mean LDL values (mmol/L) at the baseline measurement were 3.98 ± 0.94 for krill powder group and 3.86 ± 0.93 for placebo group and at the end of the intervention at day 56 3.95 ± 0.88 and 3.85 ± 0.86, mean HDL (mmol/L) as 1.49 ± 0.58 and 1.63 ± 0.34 compared to 1.48 ± 0.50 and 1.72 ± 0.43 at the end and mean triglycerides (mmol/L) 1.31 ± 0.49 and 1.29 ± 0.61 compared to 1.45 ± 0.72 and 1.24 ± 0.44 at the end of the intervention.

### Blood pressure

**Fig. 1:**
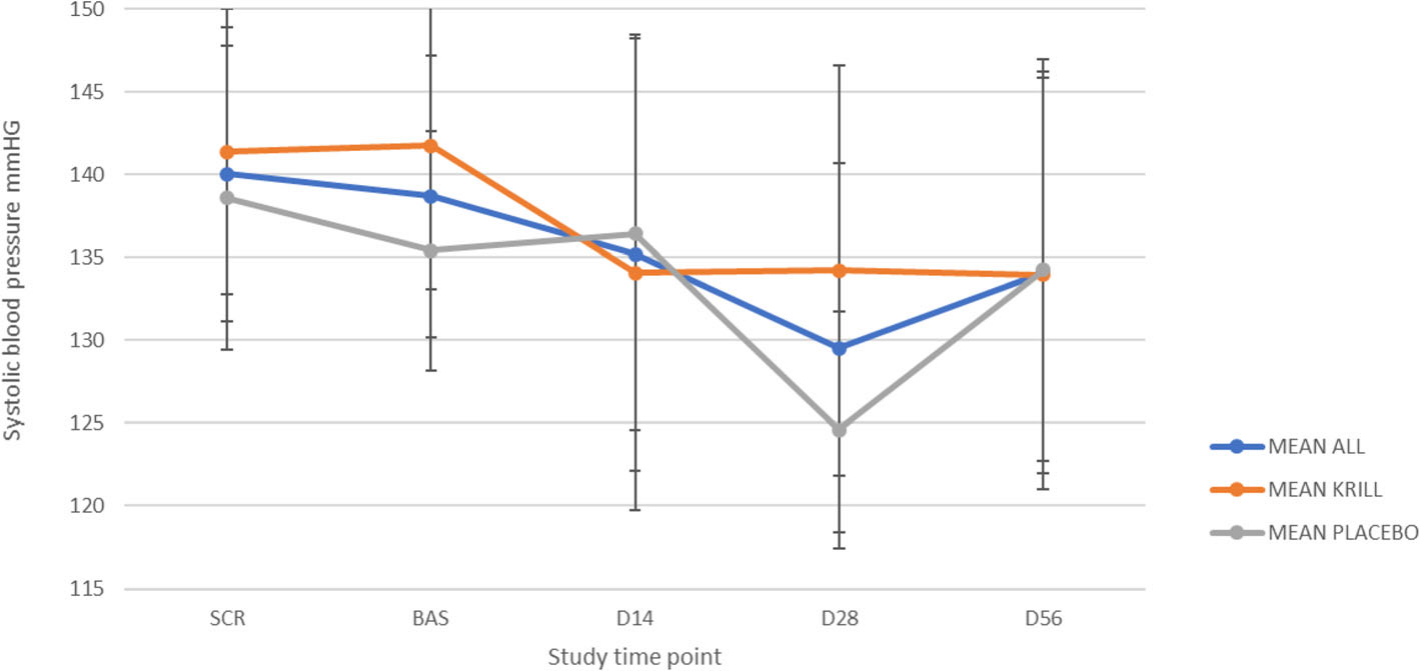
Mean diastolic blood pressure (+/-SD) during the intervention in all study subjects (*n* = 35), krill powder group (*n* = 17) and placebo group (*n* = 18)

**Fig. 2:**
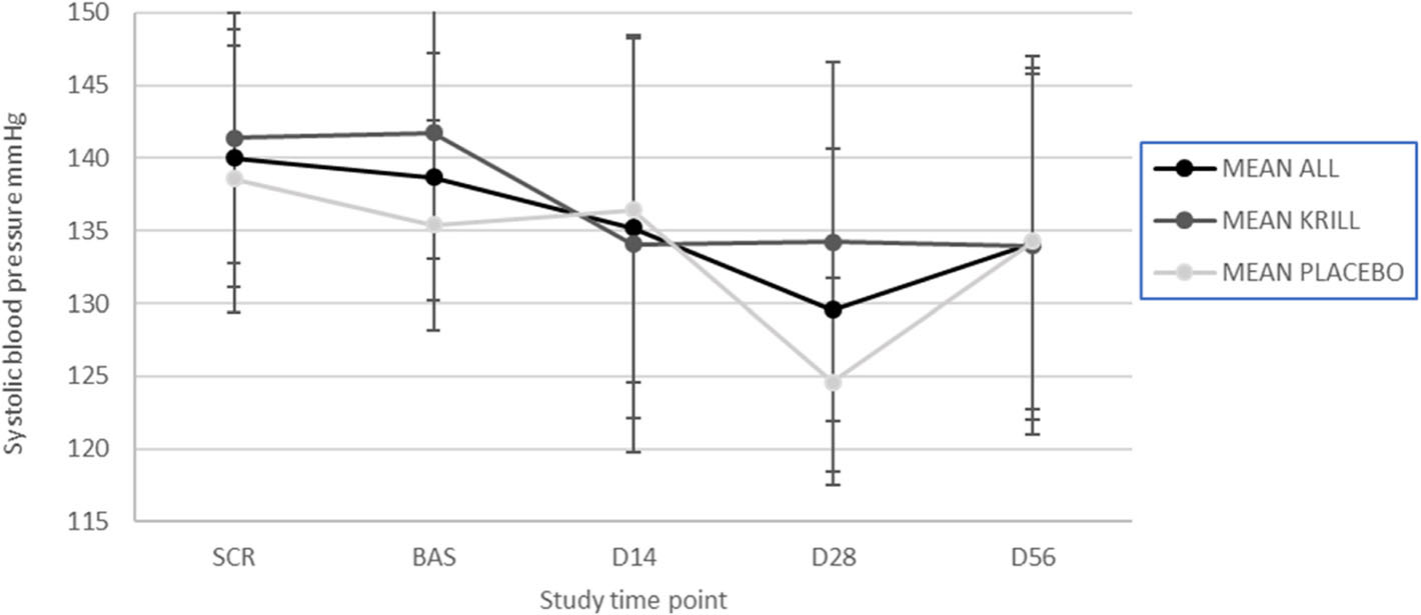
Mean systolic blood pressure (+/-SD) during the intervention in all study subjects (*n* = 35), krill powder group (*n* = 17) and placebo group (*n* = 18)

No consistent change in either the diastolic or the systolic pressure was observed in either of the groups during the intervention (Figs. 1 and 2). No statistically significant change was observed in either of the groups for diastolic pressure during the intervention (*p* = 0.09 for krill powder and 0.80 for placebo), but there was a significant decrease in systolic pressure at the end of the intervention in krill powder group (*p* = 0.0057). However, no significant difference was observed when comparing the two groups to each other (*p* = 0.12).

## Discussion

The results show that krill powder is well tolerated and safe to use in therapeutic doses. The number of reported adverse events was higher in the placebo group than in the krill powder group and only one of the reported AEs in the krill powder group was considered to be definitely related to the treatment. None of the AEs in either of the groups were severe. There were no significant changes in the hematological or clinical chemistry values either. There was sporadic deviation in liver values (ALT and AST) at one timepoint, but these elevated AST and ALT values were considered occasional findings as during other visits they remained within reference values or values were not replicated in retest (AST). This is also in line with secondary results gained from the few previously done clinical studies with krill oil, where no significant changes in hematological values nor any notable adverse events were reported during the treatment [17, 18].

Somewhat surprisingly, no significant difference was noted in any of the lipid values during the intervention between the groups. Previous studies with krill oil have indicated that when used in similar or even lower doses, positive changes in lipid values can be achieved. The other possible explanation could also be the differences between the pharmacokinetic profiles and weaker bioavailability of krill oil used in those studies and krill powder used in this study. In a study by Berge et al., significant reduction of serum triglycerides was noted without any change in LDL values [18]. Also, in a study by the same group [17], a significant change was noticed after 12 and 24 weeks of administration 4 g of krill powder to overweight subjects. However, it is quite well established that doses 0.5-5 g/day of EPA and DHA does not necessary reduce LDL cholesterol in normolipidemic, but triglycerides are reduced in subjects with elevated triglyceride concentrations [19–21] with krill oil. In case of other study by Berge et al. [17], performed with a similar dose of krill powder, the longer duration of intervention might explain why in that study significant differences were found. There were a couple of outliers in the Krill group whose triglycerides increased markedly and this could partly explain the increase in the variation during our trial. Thus, no consistent lowering trend for triglycerides could be seen. Another reason could be that the total dose of n-3 fatty acids gained via krill powder was simply too low (240 mg) in this case to generate reduction in serum triglycerides or the duration of the treatment was too short. Previous studies have shown that 2–4 g /day of EPA and DHA are needed to gain hypolipidemic effect in normolipidemic person [19].

The effects of krill powder on blood pressure in this study were inconclusive. Contrary to previous studies done with marine peptides or lipids on blood pressure, there was no significant change in diastolic pressure [8, 13]. These findings may be due to the high level of intrasubject variability of the blood pressure values during the follow-up period. However, krill powder seemed to have a lowering effect on systolic pressure that was noted at Day 14, 28 and 56 as statistically significant change when comparing krill powder to the baseline. However, blood pressure reduced also in Placebo group (at 28 day) and thus no significant difference could be found, when comparing krill powder group to placebo group. These results might indicate, that krill powder has a lowering effect on at least systolic blood pressure, but it cannot be ruled out that reduction was related to accommodation to blood pressure measurement via replicated measurements. Study was sufficiently powered to detect differences in AE reporting, number of observations were in agreement with initial power calculation even if the sample size remained smaller than planned. However, study was not sufficiently powered to see mild effects in metabolic parameters. Therefore, it would be interesting to confirm the effect of krill powder on blood pressure with a larger population to see if the lowering effect would be consistent and statistically significant, and to test different dosages of krill powder, as in some previous studies it has been noted that the lowering effect of marine peptides and lipids on blood pressure may be dose dependent [13].

## Conclusions

According to the results collected from the diaries of the participants, numerically more adverse events were reported in the placebo than in the krill powder group, but no serious adverse events were reported during the study in either of the groups. Mild gastrointestinal symptoms such as flatulence and heartburn were the most common class of symptoms possibly attributed to the test product use. There were no differences in measured haematology variables between the groups and liver and kidney enzymes remained stable except few occasional high values of AST and ALT detected both at baseline, that were occasional findings since during other visits values remained within reference values or values were not replicated in retest. Somewhat surprisingly, no significant difference was seen in the blood pressure or lipid values at the end of the intervention between the groups despite of the slight the tendency for reduction in systolic blood pressure with krill powder. These results seem to indicate that using krill powder as a source for EPA and DHA is safe in therapeutic dose and the risk of adverse events, let alone serious ones, is low.
